# Enhanced Semantic Information Transfer of Multi-Domain Samples: An Adversarial Edge Detection Method Using Few High-Resolution Remote Sensing Images

**DOI:** 10.3390/s22155678

**Published:** 2022-07-29

**Authors:** Liegang Xia, Dezhi Yang, Junxia Zhang, Haiping Yang, Jun Chen

**Affiliations:** College of Computer Science and Technology, Zhejiang University of Technology, Hangzhou 310023, China; xialg@zjut.edu.cn (L.X.); 2112012182@zjut.edu.cn (D.Y.); 2111912201@zjut.edu.cn (J.Z.); 2112012072@zjut.edu.cn (J.C.)

**Keywords:** domain adaptation, multi-class semantic edge detection, deep learning, high-resolution remote sensing

## Abstract

Edge detection of ground objects is a typical task in the field of remote sensing and has advantages in accomplishing many complex ground object extraction tasks. Although recent mainstream edge detection methods based on deep learning have significant effects, these methods have a very high dependence on the quantity and quality of samples. Moreover, using datasets from other domains in detection tasks often leads to degraded network performance due to variations in the ground objects in different regions. If this problem can be solved to allow datasets from other domains to be reused, the number of labeled samples required in the new task domain can be reduced, thereby shortening the task cycle and reducing task costs. In this paper, we propose a weakly supervised domain adaptation method to address the high dependence of edge extraction networks on samples. The domain adaptation is performed on the edge level and the semantic level, which prevents deviations in the semantic features that are caused by the overgeneralization of edge features. Additionally, the effectiveness of our proposed domain adaptation module is verified. Finally, we demonstrate the superior edge extraction performance of our method in the SEGOS edge extraction network in contrast to other edge extraction methods.

## 1. Introduction

The extraction of ground objects from high-resolution remote sensing images plays an important role in many fields, such as urban planning and change detection [1,2]. In addition, due to the characteristics of high-resolution remote sensing images, such as dense information and many interference factors [3], many ground object extraction methods have poor performance on the edge. Methods such as semantic segmentation need more complex designs to meet the edge accuracy requirements of the task, especially for complex tasks such as the extraction of dense objects [4,5] or the segmentation of multi-category objects [6]. The edge detection method concentrates more on the quality of the edge and can also help other methods obtain better-quality edges when combined [7,8]. However, recent mainstream ground extraction methods are based on data-driven deep learning methods. Reusing samples is difficult because of the differences in the domains, and, as a result, each task needs to create a new sample set, which greatly increases the task cost and timeframe.

To reduce the need for the number of samples, domain adaptation methods reuse samples that are not in task domains by reducing the distribution shift between different task domains [9]. In these methods, the task domain with labels that can fully train the model is usually known as the source domain, and the task domain with no labels or only a few labels is called the target domain. Due to the differences in the distribution of the ground objects, shooting angles, lighting effects, and image sources between the source and target domains in the remote sensing field, simply using the source domain samples as the train samples in the target domain task will greatly reduce the extraction performance of existing methods [10]. By reducing the distribution shift caused by these differences, the source domain samples can be effectively used in the target domain. Currently, unsupervised domain adaptation (UDA) has been widely used in image processing tasks such as classification and semantic segmentation. There are also methods that propose semisupervised domain adaptation (SSDA) methods [11,12] to further improve this effect. However, as far as we know, there is still no method to use domain adaptation in edge detection.

Currently, the mainstream domain adaptation methods are adversarial learning [9,13,14,15] and self-training learning [16,17,18,19,20]. We found that after directly introducing adversarial learning into edge detection, the effect was not significantly improved. The reason is that the semantic edge contains low-level edge features with strong generalization and high-level semantic features with obvious differences. The edge features after adaptation will lead to more obvious differences in the semantic features; thus, the network will detect the edges that do not belong to the target objects. The self-training learning uses pseudo-labels to expand the number of samples in the target domain [11]. However, edge detection requires high accuracy and closure, and the pseudo-labels are quite different from the real labels, which is more difficult to solve in multi-class edge extraction. Therefore, we propose a weakly supervised domain adaptation (WSDA) method based on adversarial learning to reduce the difference in semantic information between two domains.

We used one extractor and two discriminators for adversarial learning. The extractor is used to detect edges and output an edge strength map (it describes the confidence of the predicted edge). Then, the first discriminator inputs the edge strength map to perform domain adaptation on the edge level. The second discriminator inputs the mean map of the ground truth and the edge strength map to perform domain adaptation on the semantics level. In addition, during training, we set different weights for each part, especially the dynamic parameters used inside the extractor. In the early stage of training, overfitting caused by too few samples in the source domain is prevented, and the training weight of the source domain is increased so that the network can fully learn enough edge features. In the later stage of training, the training weights of the source domain are reduced so that the source domain samples are fine-tuned to provide accurate semantic features. As a result, our method achieves its performance with few target domain samples compared with fully supervised edge extraction networks that use sufficient source domain samples.

The main contributions of this method are as follows:

1. Domain adaptation is applied to edge detection for the first time.
2. A weakly supervised domain adaptation method for high-resolution remote sensing object edge detection is proposed. This method performs domain adaptation at the edge level and the semantic level to reduce the difference between two domains in the edge detection network.

The rest of the paper is organized as follows. Section 2 reviews the work related to edge detection and domain adaptation. Section 3 introduces the edge detection network structure that is proposed in this paper with a thorough introduction of the weakly supervised domain adaptation method. Section 4 presents the dataset, experimental design, experimental results, and some discussions about the experiments. Section 5 summarizes the paper and makes some suggestions for future work.

## 2. Related Work

This section introduces edge detection and domain adaptation, respectively.

### 2.1. Edge Detection

Previous edge detection methods use low-level features to determine whether each pixel of an image belongs to a contour. Convolving the image with a local filter detects the pixel with the highest gradient magnitude in its local neighborhood as an edge. To extract discontinuous features that are generated by step edges, linear filters such as Canny [21] and Sobel [22] are used [23]. However, for complex images, such as images with a complex texture, low contrast, and high information density, these methods cannot generate effective boundaries because they only detect the local features of edges. Many methods [24,25] assist edge detection by introducing semantic-level information. They are improved compared with previous pixel-based methods but still have limited generality and need to adjust the edge detection strategy based on the dataset.

Due to the success of CNNs, many edge detection methods based on deep learning have been proposed. Liu, Cheng, et al. [26] combined the hierarchical features of all convolutional layers in VGG16 into a holistic framework, which enables the network to learn multi-scale information that is both low-level and object-level and better detect edge information. He, Zhang, et al. [27] proposed a bidirectional cascade network structure so that the output of each layer is supervised by edge labels of a specific size, which can force each layer to focus on a specific scale and then fuse the results of different scales that are output by each layer. Poma, Riba, et al. [26] proposed a dense extreme inception network that avoids lost edges in deeper layers by generating thin edge maps. Su, Liu, et al. [28] combined traditional edge detection operators with convolutional operations to make modern CNNs more focused on processing edge gradient information in the image, thereby detecting edges with semantic information faster and improving the accuracy of edge detection. However, these deep learning-based methods focus more on obtaining better edge detection performance with sufficient samples and still have difficulty achieving satisfactory results when the number of samples is too small.

The development of edge detection methods based on deep learning has brought more solutions to the information extraction of high-resolution remote sensing images. Wei et al. [29] used the U2-net [30] semantic segmentation model to detect building edges and replaced the original loss function with a multi-class cross-entropy loss function to directly generate a binary map with edges and backgrounds. Xia et al. [31] proposed a building edge detection method that uses Faster R-CNN [32] to detect the bounding box of the building and uses the bounding box to assist in the repair of the broken line to completely extract the building outline. These methods are still fully supervised methods, and the labeling of high-resolution remote sensing image samples is more complicated than that of natural images; thus, research into edge extraction methods with a small number of samples is more necessary.

### 2.2. Domain Adaptation

Domain adaptation methods reuse the labeled samples in the source domain by aligning the distribution offset between the source domain data and the target domain data, which reduces the high dependence of deep learning methods on the number of labeled samples in the target domain.

One of these ideas is adversarial learning [9,13,14,15], which uses an extractor to extract the same features from different task domains, and a discriminator to identify data from different task domains. The two play against each other, thereby reducing the domain differences between the two domains. The typical method is AdaptSegNet [14], which is the first domain adaptation method to adopt adversarial learning in the output space. Based on this, Vu, Jain, et al. [33] proposed a method for adversarial entropy, using entropy as a measure for unsupervised adversarial learning. Although these two methods do not use the target domain samples, there are still obvious noises in the results, which lead to blurred edges. ASS [34] is the first work on SSDA for semantic segmentation and performs semantic adaptation pixel by pixel. However, the pixel-by-pixel adversarial method ignores the correlation between edge pixels and cannot be effective in edge extraction. Such methods can theoretically be applied to most tasks, but the strong generalization of edges in edge detection tasks will lead to more incorrect detections. Therefore, adjusting the mainstream methods used in semantic segmentation is necessary.

Another idea is self-training [16,17,18,19,20], which predicts the target domain data through a network that is trained by the source domain data. Zou et al. [20] proposed a typical self-training method that screened out reliable pseudo-labels according to the confidence and then added prior spatial information to assist the training of the target domain to ensure the reliability of fine-tuning that uses pseudo-labels. However, the threshold for selecting pseudo-labels affects the effect of the self-training methods, and using a constant threshold for different training times is not a good strategy. Zheng, Yang, et al. [19] proposed a method to generate a dynamic threshold through the variance in the result so that each round of the self-training process can generate higher-quality pseudo-labels. Yu, Liu, et al. [18] proposed a three-level feature alignment method to match global, local, and instance features between the source and target domains, respectively, and generate more accurate pseudo-labels through multi-level feature alignment to improve the effect. These methods try to generate more accurate pseudo-labels to reduce the impact of missing samples in the target domain. Such methods can achieve better results for simple tasks such as classification tasks or nonedge-dominated complex tasks such as semantic segmentation. However, for the task of edge detection, which has high requirements for edge accuracy, edge closure, and other indicators, there are obvious differences between pseudo-labels and real labels; as a result, ensuring the effectiveness of such methods in edge detection methods is difficult.

Many methods apply domain adaptation to the high-resolution remote sensing image information extraction field. Song et al. [35] designed a subspace alignment module to add to the CCN model, which alleviated the domain distribution discrepancy and somewhat solved the problem of different domain samples in scene classification. Yao et al. [36] proposed a weakly supervised domain adaptation method by utilizing adversarial entropy, which addresses the domain gap problem in building semantic segmentation by using an adversarial entropy strategy and a self-training strategy. However, these methods for nonedge-dominated tasks, such as classification tasks and semantic segmentation tasks, have less reference to edge extraction. As far as we know, there is still no effective domain adaptation method in edge detection.

## 3. Methodology

Our method uses two datasets in different regions, in which the semantic edges of the same type of ground objects are labeled. The source domain dataset contains a large number of samples; the target domain dataset contains a small number of samples. In our method, $D_{s}={\left\{X_{s\_i},Y_{s\_i}\right\}}_{i=1}^{N_{s}}$ is used to denote $N_{s}$-labeled source domain samples, and $D_{t}={\left\{X_{t\_i},Y_{t\_i}\right\}}_{i=1}^{N_{t}}$ is used to denote $N_{t}$-labeled target domain samples. Among them, $X_{s\_i}$ and $X_{t\_i}$ are high-resolution remote sensing images of W×H×4 and can be collectively referred to as *X*; $Y_{s\_i}$ and $Y_{t\_i}$ are both gray-value map labels of W×H×1 and can be collectively referred to as *Y*.

Adversarial learning is performed between the edge detection module and the two adaptation modules, each of which is continuously enhanced. If the features generated by the edge detection module can confuse the identification of the adaptation module, the distribution of the features extracted from the source domain image and the target domain image is considered consistent.

**Figure 1:**
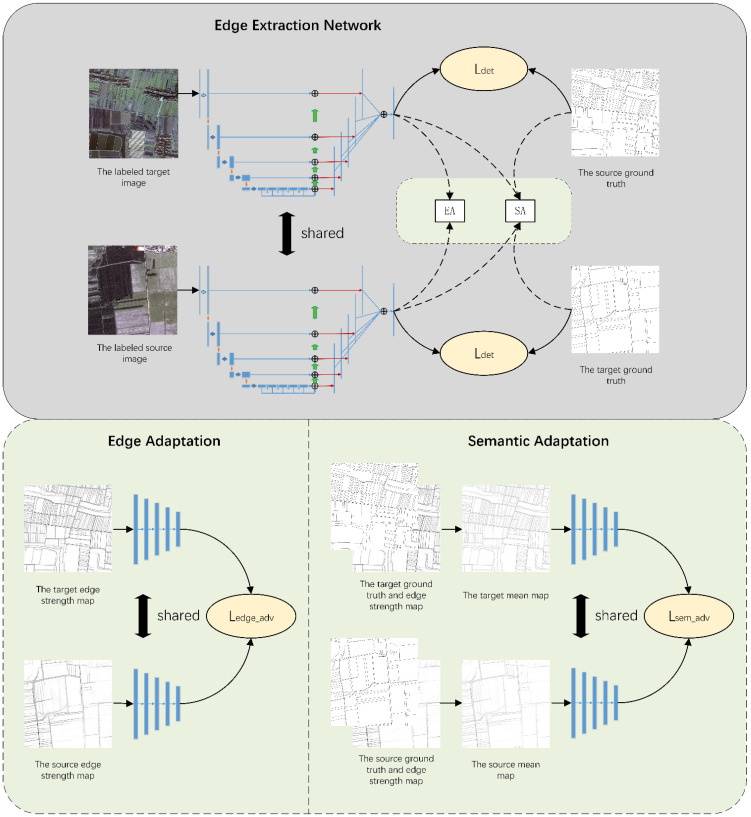
Algorithmic overview. The network can be divided into the following three modules: the edge detection module (ED), the edge adaptation module (EA), and the semantic adaptation module (MA). ED is used as an extractor, which is the main body of the network to detect the semantic edge of the target object. EA and MA are used as the discriminators. The EA takes the edge strength map output by the edge detection module as the input to perform domain adaptation on the edge level. MA takes the mean map of the edge strength map and the ground truth as the input to perform domain adaptation on the semantic level.

Each part of the network will be described in detail below. Figure 1 shows an overview of the proposed algorithm.

### 3.1. Edge Detection Module (ED)

For the edge detection task of high-resolution remote sensing images, connectivity is also one of the important indicators to evaluate the quality of the edge. The edges of objects such as cities and roads are often widely distributed, so they should have a large receptive field size to retain detailed spatial information. Therefore, SEGOS [37], which is modified based on D-LinkNet [38], is chosen for this paper. D-LinkNet was originally used to extract the road centerline, and its network structure can be divided into the following three parts: the encoder, central part, and decoder. The encoder part is composed of ResNet34. To cope with the characteristics of the abovementioned edge features, D-LinkNet adds a central part that is composed of dilated convolution for multi-resolution spatial information perception, which can expand the receptive field without reducing the resolution of the feature maps. In the decoder part, the transposed convolutional layer is used for upsampling to restore the size of the feature map to the original image size. SEGOS retains the core structure of D-LinkNet, sets a side output layer at each stage to control the edge loss, and merges the multi-scale side output layer into the output layer. By predicting multi-scale data, the data are merged into the output edge map to ensure the accurate semantic edge detection of objects.

After inputting $X_{s\_i}$ and $X_{t\_i}$ into G, the predicted edge strength maps $P_{s}$ and $P_{t}$ can be obtained, which can be collectively referred to as P or the input of EA. Taking the mean value of $Y_{s\_i}$ and $P_{s}$ can obtain the mean value map $A_{s}$, and taking the mean value of $Y_{t\_i}$ and $P_{t}$ can obtain the mean value map $A_{t}$, which can be collectively referred to as A or the input of SA.

By considering that the distribution of the edge pixels and nonedge pixels is extremely unbalanced, the class-balanced cross-entropy loss function is usually used, and the influence of this unbalanced distribution on the network training process is reduced by introducing the edge scale parameter λ. The loss function formula is as follows:

(1) $$L_{cbce}=-\lambda \underset{H,W}{\sum }\log\left({\hat{y}}_{j}\in \left|y-\right|\right)-\left(1-\lambda \right)\underset{H,W}{\sum }\log\left({\hat{y}}_{j}\in \left|y+\right|\right)$$

The edge detection network outputs an edge strength map to describe the confidence in predicting object boundaries. However, edge pixels are not independent, and cross-entropy loss does not take into account the continuity of the edges. Therefore, the mean-square error (MSE) loss is added to mitigate this effect:

(2) $$L_{mse}=\underset{H,W}{\sum }{\left({\hat{y}}_{j}-y_{j}\right)}^{2}$$

Therefore, the edge detection loss is the weighted sum of the above loss functions, and *α* and *β* are their weights. The loss function formula is as follows:

(3) $$L_{det}=\alpha L_{cbce}+\beta L_{mse}$$

### 3.2. Edge Adaptation Module (EA)

The module consists of multiple convolutional layers whose number of channels, kernel, and stride can be adjusted according to the resolution of the remote sensing imagery. Except for the last layer, each convolutional layer is followed by a weakly ReLU [39], which finally outputs a vector of size 1*1. We implement the labeling of the source domain as 0 and the target domain as 1. Then, the edge strength map output is input by the edge detection module for supervised training. The marginal distribution loss function uses binary cross-entropy (BCE) loss, which can be expressed as:

(4) $$L_{edge\_adv}=-logEA\left(P\right)$$

However, since the edge strength map focuses more on the edge information, the semantic information of the semantic edge is weakened. Therefore, only using the edge adaptation module will lead to more generalization of the transferred edge feature, which will increase confidence in the nontarget object edges and result in no significant changes. Therefore, we should also consider how to better transfer the semantic features.

### 3.3. Semantic Adaptation Module (SA)

Considering that weakly supervised domain adaptation has a small number of target domain sample labels in contrast to unsupervised domain adaptation, the ground truth contains correct semantic information, so the edge strength map can be combined with the ground truth. First, their mean map is obtained and then input into the network with the same structure as the edge adaptation module; thus, more semantic features can be obtained during the adaptation process. The semantic distribution loss function can be expressed as:

(5) $$L_{sem\_adv}=-logSA\left(A\right)$$

### 3.4. Adversarial Learning Process

To make the edge detection module and the adaptation module perform adversarial learning, $X_{s\_i}$ is first input to the ED, $P_{s}$ is obtained, and the source domain edge detection loss function $L_{s\_det}$ is calculated. Then, input $X_{t\_i}$ into the ED to obtain $P_{t}$, and calculate the target domain edge detection loss function $L_{t\_det}$. Moreover, $P_{s}$ and $P_{t}$ are input into the EA to obtain their respective edge loss functions $L_{s\_\mathrm{edge}\_\mathrm{adv}}$ and $L_{t\_\mathrm{edge}\_\mathrm{adv}}$. Then, the mean maps $A_{s}$ and $A_{t}$ generated by *P* and *Y* are input into the *SA* to obtain their respective semantic distribution loss functions $L_{s\_\mathrm{sem}\_\mathrm{adv}}$ and $L_{t\_\mathrm{sem}\_\mathrm{adv}}$.

Each module has its own weight, and there is also a dynamic parameter δ inside the edge detection module to adjust the role of the samples of the two domains in the network training process. The weight of the source domain samples is increased in the early stage of network training to prevent overfitting caused by too few samples in the target domain. At the end of network training, the weight of the source domain samples is reduced to prevent them from providing too much inaccurate semantic information, and the network is fine-tuned to detect more accurate semantic edges of the target domain.

Thus, for ED, the loss function is:

(6) $$L_{DE}=\delta L_{s\_det}+(1-\delta )L_{t\_det}+L_{s\_\mathrm{edge}\_\mathrm{adv}}+L_{t\_\mathrm{edge}\_\mathrm{adv}}+L_{s\_\mathrm{sem}\_\mathrm{adv}}+L_{t\_\mathrm{sem}\_\mathrm{adv}}$$

For EA, its loss function is:

(7) $$L_{EA}=L_{s\_\mathrm{edge}\_\mathrm{adv}}+L_{t\_\mathrm{edge}\_\mathrm{adv}}$$

For SA, the loss function is:

(8) $$L_{SA}=L_{s\_\mathrm{sem}\_\mathrm{adv}}+L_{t\_\mathrm{sem}\_\mathrm{adv}}$$

Based on our goal of adversarial learning, we optimize the following min–max criterion:

(9) $$\underset{ED}{\max}\underset{EA,SA}{\min}\left(L_{DE},\;L_{EA},\;L_{SA}\right)$$

The goal is to minimize edge detection loss while maximizing the probability that the target domain predictions are regarded as the source domain predictions.

## 4. Experiment and Results

In this section, we present the experimental details and the results.

### 4.1. Experimental Details

#### 4.1.1. Data Sets

The data sets selected in this paper are two sets of multi-category semantic edge datasets, both of which are data sets that were created in our research laboratory. The first set of data sets was produced through the GF2 PMS imagery in Yangyuan County, Zhangjiakou City, Hebei Province. Each image is 1000 pixels × 1000 pixels × 4 channels, for a total of 500 images. Edges of water, crops, fruit trees, forests, buildings, grass, and roads are labeled in the sample. The second set of data sets was produced through the GF2 PMS imagery in Jiashan County, Jiaxing City, Zhejiang Province. Each image is 1000 pixels × 1000 pixels × 4 channels, for a total of 309 images. The edges of the same aforementioned objects are labeled in the samples. A total of 103 samples were selected from each of the two sets of data for testing, and the remaining samples were used for training.

**Figure 2:**
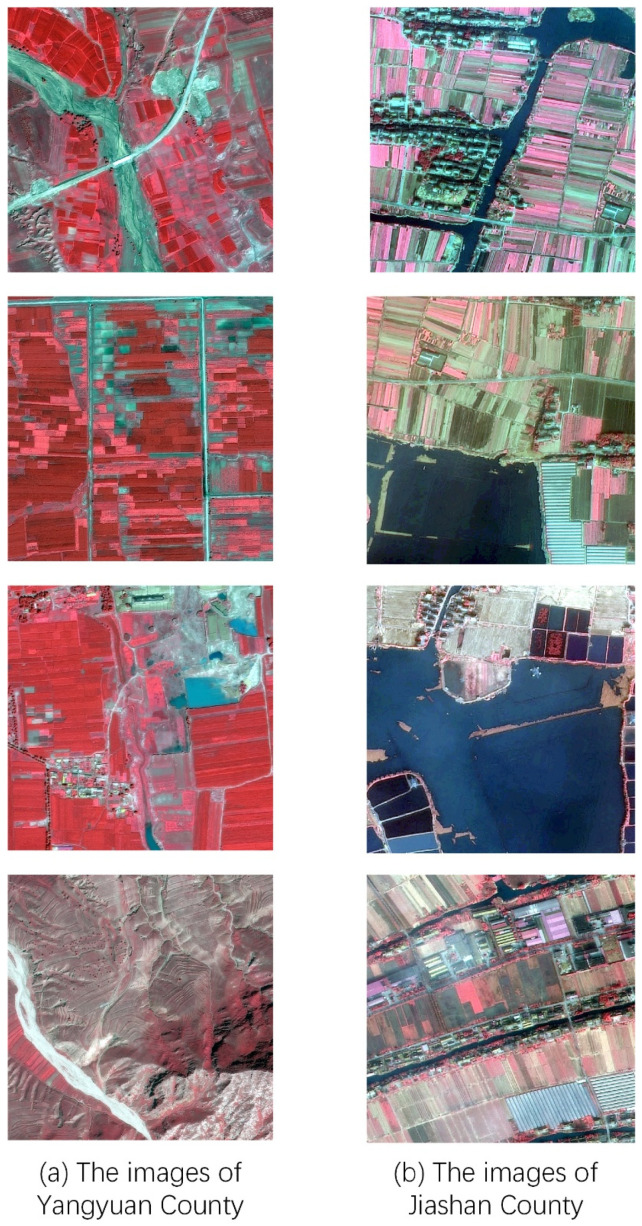
Sample images between (**a**) Yangyuan County and (**b**) Jiashan County. The distance between the two selected regions is approximately 2000 km. Yangyuan County is in a basin with a wide distribution of mountainous areas. Jiashan County is in a plain, with a large proportion of water. There are obvious differences in the topography and landforms of the two places.

As shown in Figure 2, the features and distribution of the two regions are significantly different. The samples of the source domain and the target domain were labeled by different teams at different times. Therefore, there are also certain differences in the labeling standards. Since the domain differences are obvious, the effectiveness and generality of our method can be fully verified.

#### 4.1.2. Implementation Details

We implemented our method using the PyTorch [40] deep learning framework. All experiments are performed on a single NVIDIA 3090 graphics card with 24 GB of memory. Our models are all trained using the Adam optimizer [41] with a learning rate of $10^{-4}$; the learning rate of the ED module is reduced by a factor of 10 every quarter epoch. In the ED module, the weights of the source domain and the target domain are $\frac{1}{epoch}$ and $\left(1-\frac{1}{epoch}\right)$, respectively. The weights of the other adaptation modules are all 0.001.

#### 4.1.3. Compared Methods and Evaluation

Our method is compared with AdaptsegNet, AdvEnt, and ASS for domain adaptation and with BDCN, DexiNed, and SEGOS for edge detection. The compared methods use optimal parameters for training. For the domain adaptation methods, the loss is the same as the loss function used in our method. The ground truth of the target domain samples is not used in the baselines and unsupervised methods. For the edge detection methods, since they do not use multi-domain samples, they are used for training: only the source domain samples, only the target domain samples, and mixing the two domain samples.

The evaluation is performed using the optimal dataset scale (ODS) and optimal image scale (OIS) proposed by HED [42], which have been widely used in edge detection methods [27,43,44]. The edge strength map needs to first be processed by using the standard nonmaximum suppression (NMS) method. Then, the same threshold is set for all processing results; that is, a fixed threshold is selected to be applied to all results so that the F1-score on the entire dataset is the largest, and the average value of the F1-score is used as the ODS. Different thresholds are selected for each processing result to maximize the F1-score. Moreover, the average value of the F1-score is used as the OIS.

### 4.2. Experimental Results

In the following experiments, except for the last set of experiments, all samples in the Yangyuan training set were selected as the source domain samples, and 51 samples were selected from the Jiashan training set as the target domain samples to compare the extraction performance of the methods with a small number of target domain samples.

The last set of experiments verifies the edge detection of our method by using different numbers of target domain samples in different transfer directions. Our method performs domain adaptations from Yangyuan County to Jiashan County and from Jiashan County to Yangyuan County. Additionally, SEGOS is trained only with samples from the target domain. All samples from the source domain will be used, and 25%, 50%, and 100% of the target domain samples will be used for training.

The results of all experiments are predicted on the test set of the target domain.

#### 4.2.1. Ablation Experiment

**Figure 3:**
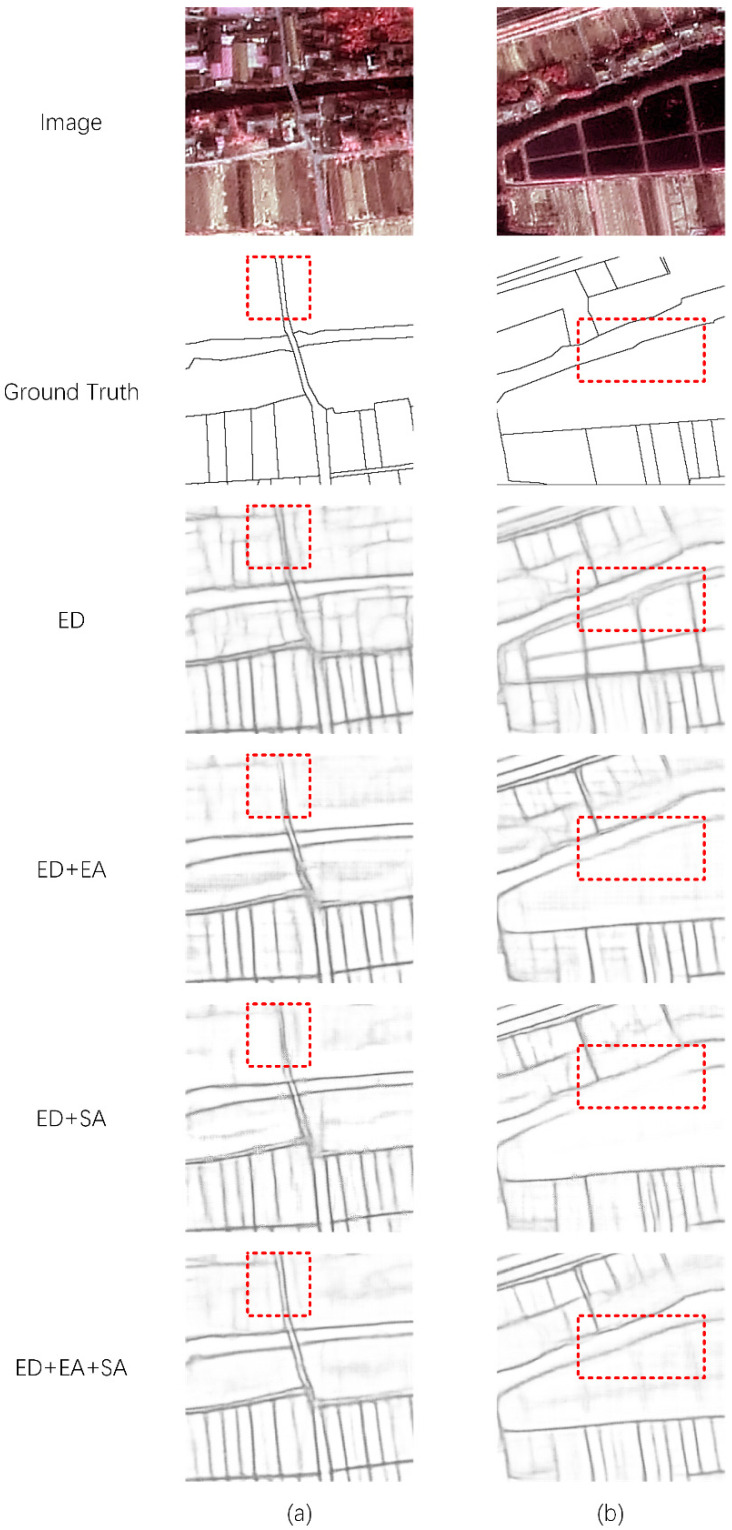
Comparison of the results of the ablation study. (**a**,**b**) are the results of different images. For each image, we show the results of the ED module, ED module with EA module, ED module with SA module, and our full method.

As shown in Table 1, better results can be obtained by using the EA module and the SA module together. Thus, every module is necessary for our method. As shown in Figure 3, we selected some representative results from the test results. We can see that the difference between the training sets of the two domains causes the ED module to detect the edges of many nontarget objects. After adding the EA module or SA module to the ED module, this problem is largely alleviated, but blurred edges are still detected in some complex areas. We combine the two so that the method can obtain satisfactory edge extraction results in these regions.

#### 4.2.2. Domain Adaptation Module Comparison

**Figure 4:**
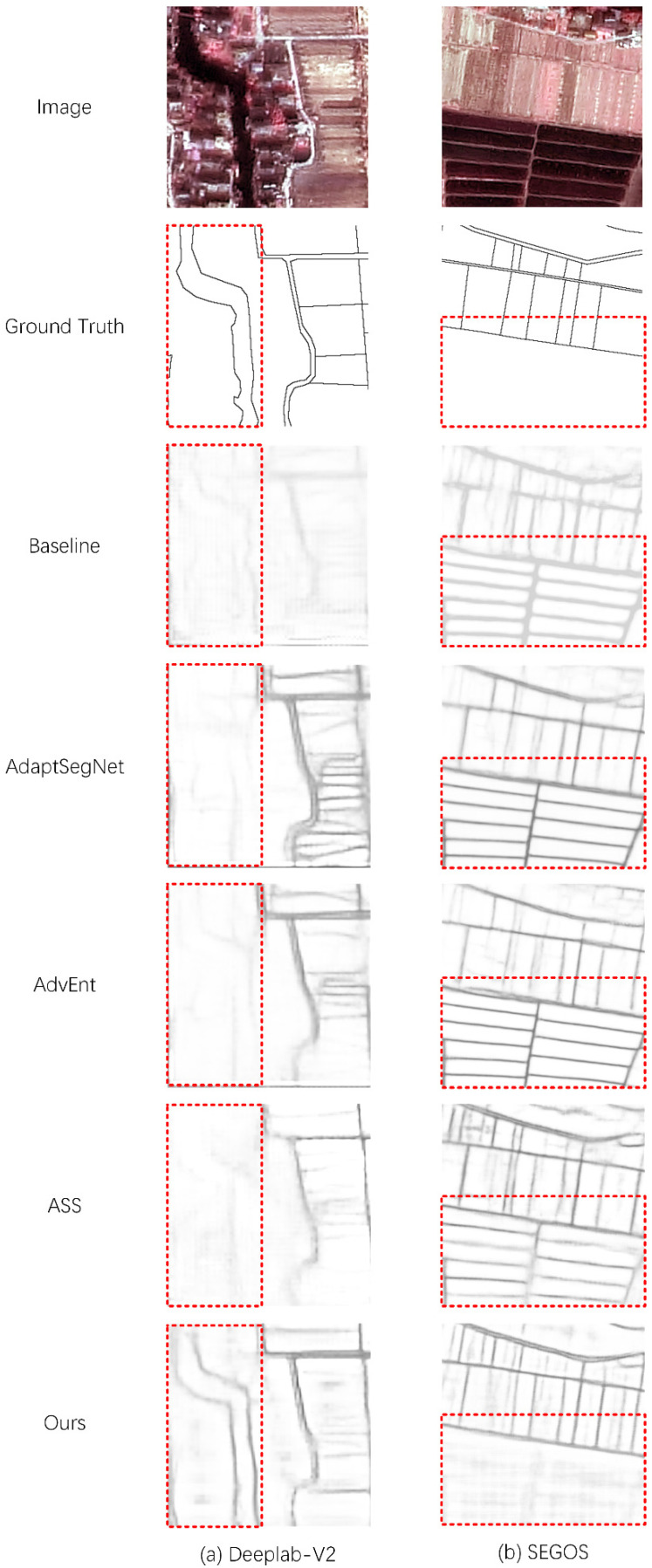
Comparison of the results of domain adaptation methods. (**a**,**b**) are the results of different edge extraction networks on the image. For each image, we show the results of baseline, AdaptSegNet, AdvEnt, ASS, and our method.

As seen in Table 2, using UDA methods for edge detection provides only a small improvement. The evaluation of ASS has been significantly improved. However, it can be observed from the results (Figure 4) that in some regions with obvious domain differences, the extraction results still retain obvious source domain semantic information. Moreover, our method has a better domain adaptation effect than other methods in these regions.

#### 4.2.3. Edge Detection Effect Comparison

As seen in Table 3, for fully supervised edge detection methods, even if the number of samples is much larger than the target domain samples, the training effect of the target domain samples is not as good. Very few samples in the target domain cause the network to be unable to train sufficiently. Moreover, mixing source domain samples and target domain samples may yield different results depending on the network structure. In contrast, ASS makes the source domain samples more stable in the target domain. Our proposed method adds the transfer of more semantic information, which has a greater improvement than the semisupervised methods.

**Figure 5:**
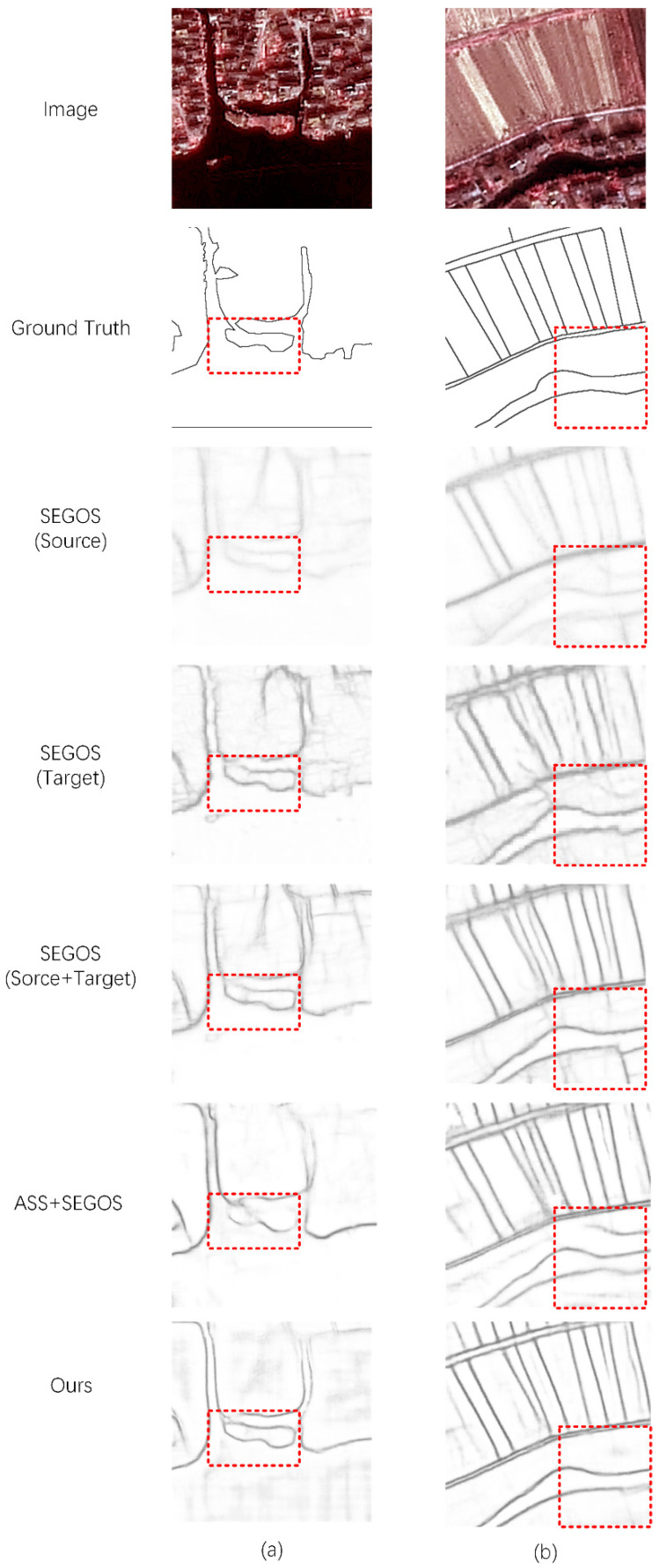
Comparison of the results of edge extraction methods. (**a**,**b**) are the results of different images. For each image, we show the results of SEGOS trained with the source domain dataset, SEGOS trained with the target domain dataset, SEGOS trained with a mixture of the two domain datasets, AAS, and our method.

Figure 5 shows the test results of SEGOS (the best-performing fully supervised edge detection network), ASS, and our method. It can be observed that the performance of fully supervised edge detection is consistent with the above conclusions. ASS using pixel-wise semantic adaptation cannot perform effectively in edge detection. Our method achieves the best results.

#### 4.2.4. Comparison of Different Numbers of Target Domain Samples

From the experimental results of SEGOS in Table 4, the difference in the number of samples will have a significant impact on the network training, and the network cannot fully learn the edge information if the number of samples is too small. After introducing source domain samples by using our method, better training results can be obtained with the same number of target domain samples.

## 5. Discussion

This study aims to make domain adaptation more effective in edge extraction of high-resolution remote sensing images, thereby obtaining better edge extraction results using a small number of target domain samples. The effectiveness and advancement of this method are verified from multiple perspectives through four sets of experiments.

As observed from the results (Table 1 and Figure 3) of the ablation study, although the EA module and the SA module perform domain adaptation at different levels of the semantic edge, the effect is limited. The use of edge-level or semantic-level adaptation alone will bias the training of the network, resulting in erroneous detections at the edges of some complex objects. When our method uses them together to constrain the adaptive process of semantic edges, the effect is significantly improved.

We compared our method with other domain adaptation methods in different edge detection networks. The results (Table 2 and Figure 4) show that since the UDA method does not perform semantic-level adaptation in edge detection, false edges caused by domain differences can still be detected in the results. ASS uses pixel-by-pixel semantic adaptation, a method originally used for semantic segmentation that does not consider the correlation between edge pixels, resulting in more broken lines in the results. Our method introduces ground truth to perform semantic-level adaptation on the entire image, effectively solving the abovementioned semantic differences between different domains.

The comparison results (Table 3 and Figure 5) of edge detection methods show that the simple, mixed multi-domain sample strategy cannot make the source domain samples play a stable role in a fully supervised network. Using the DA method makes source domain samples effective for network training. In addition, our method combines SEGOS with multi-level adaptive modules and achieves the best results in multi-category edge detection tasks in high-resolution remote sensing images.

**Figure 6:**
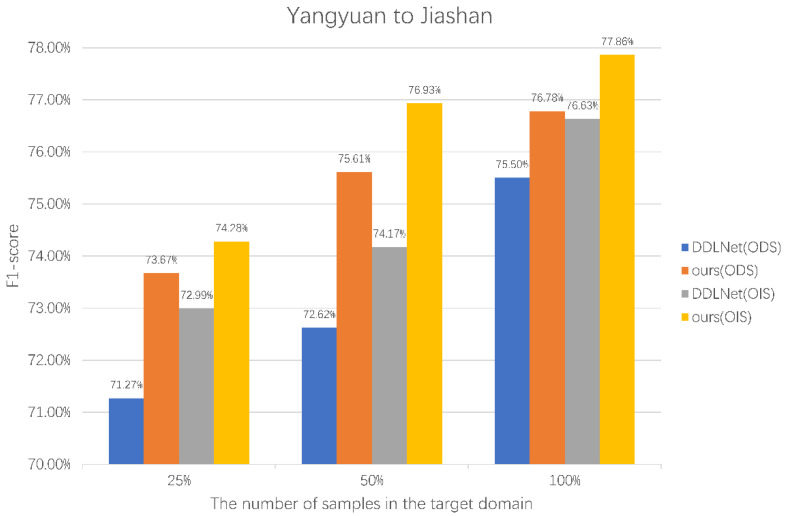
Comparison of experimental results between SEGOS and our method using different numbers of samples in Jiashan County.

**Figure 7:**
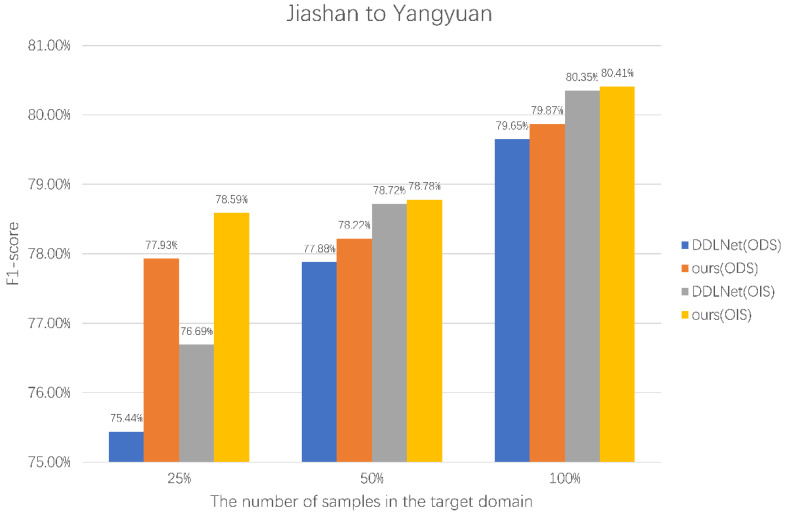
Comparison of experimental results between SEGOS and our method using different numbers of samples in Yangyuan County.

Figure 6 and Figure 7 are the comparison results of our method and SEGOS under different target domain samples. It can be observed that the introduction of source domain samples significantly improves ODS and OIS when using different numbers of target domain samples. When the target domain samples are insufficient, the improvement in this method is more obvious. This verifies that our method effectively improves the effect of edge detection methods using a small number of target domain samples by introducing source domain samples.

In summary, most of the current domain adaptation methods for semantic segmentation only perform domain adaptation at a single level. Some multi-level domain adaptation methods are also not suitable for semantic edges. The semantic edge contains low-level edge information and high-level semantic information, which easily makes the adaptive process biased and affects the training of edge extraction networks. Therefore, our method performs an adaptation process at both the edge level and the semantic level, using them together to constrain the transfer of semantic edges. At the semantic level, we further introduce ground truth to adapt the entire image so that the network is guided by more accurate semantic information. From the results, it can be seen that our method has an excellent performance in both regions with large domain differences and regions with small domain differences. The overall evaluation results are also higher than the current state-of-the-art methods in all aspects.

However, it can be observed from the experimental results that even though adversarial learning is used to improve the edge extraction performance of the network, there are still some pixels lost in the result, resulting in broken lines. If these results are used as pseudo-labels, there is a clear difference from the manually labeled samples. Therefore, the self-training method using pseudo-labels is limited by the difference between the extracted edge and the manually labeled edge and cannot play an effective role. If pseudo-labels similar to human labels can be made by broken-line repairing methods, they will go one step further to provide effective semantic information for the target domain. However, for complex tasks, such as multi-category semantic edge detection, this is still more difficult, so further research into self-training learning is needed.

## 6. Conclusions

In this paper, we apply the domain adaptation method to edge detection for the first time and reduce the new task domain’s high dependence on the number of samples through weakly supervised domain adaptation. First, by considering the distribution characteristics of ground objects in remote sensing images, we choose SEGOS as the edge detection network. By understanding that the UDA methods lack correct semantic information guidance in the edge detection, we add a small number of target domain samples to make full use of the ground truth of the target domain to provide the correct semantic information. Simultaneously, domain adaptation is performed on the edge level and the semantic level, and dynamic parameters are introduced to adjust the influence weights of the source domain samples on the network training in different training stages. Finally, we conduct sufficient experiments on high-resolution remote sensing data sets that were developed in our research laboratory. The effectiveness of our proposed domain adaptation module is verified. Furthermore, we demonstrate the superior edge extraction performance of our method in the SEGOS edge extraction network in contrast to other edge extraction methods. In addition, if we can further study how to repair the broken lines on the edges, the self-training learning will further improve the edge extraction effect using a small number of target domain samples.

## Figures and Tables

**Table 1:** Accuracy comparison among the methods with different modules.

Methods	ODS	OIS
ED	71.32%	72.65%
ED+EA	72.34%	73.13%
ED+SA	73.15%	74.28%
ED+EA+SA	**73.67%**	**74.28%**

**Table 2:** Accuracy comparison among different domain adaptation methods based on the (a) Deeplab-V2 [45] model and (b) SEGOS model.

Type	Methods	(a) Deeplab-V2		(b) SEGOS	
		ODS	OIS	ODS	OIS
No DA	Baseline	63.64%	65.09%	65.18%	67.13%
UDA	AdaptSegNet	65.59%	67.25%	67.75%	69.72%
	AdvEnt	65.89%	67.35%	67.34%	68.88%
SSDA	ASS	68.89%	70.17%	72.14%	73.19%
WSDA	Ours	**69.12%**	**71.13%**	**73.67%**	**74.28%**

**Table 3:** Accuracy comparison among different edge detection methods.

Type	Methods	Samples	ODS	OIS
Supervised	BDCN	Source	47.77%	48.54%
		Target	48.26%	48.27%
		Source + Target	49.11%	51.27%
	DexiNed	Source	54.45%	54.68%
		Target	69.24%	69.63%
		Source + Target	58.54%	58.65%
	SEGOS	Source	67.66%	69.50%
		Target	71.27%	72.99%
		Source + Target	72.06%	73.40%
SSDA	AAS+SEGOS	Source + Target	72.14%	73.19%
WSDA	Ours	Source + Target	**73.67%**	**74.28%**

**Table 4:** Accuracy comparison of SEGOS and our method using different numbers of target domain samples.

Methods	Target Domain	Number of Samples	ODS	OIS
SEGOS	Jiashan	25%	71.27%	72.99%
		50%	72.62%	74.17%
		100%	75.50%	76.63%
	Yangyuan	25%	75.44%	76.69%
		50%	77.88%	78.72%
		100%	79.65%	80.35%
Ours	Jiashan	25%	73.67%	74.28%
		50%	75.61%	76.93%
		100%	76.78%	77.86%
	Yangyuan	25%	77.93%	78.59%
		50%	78.22%	78.78%
		100%	79.87%	80.41%

## Data Availability

The paper provides the database used in the current study at baiduyun (https://pan.baidu.com/s/1fl4AgxPaxi-W6TSTJdkpmQ?pwd=xedc; extraction code: xedc, accessed on 10 June 2022) and the python code available online at GitHub (https://github.com/Wind-song/ESIT, accessed on 10 June 2022).
